# Editorial: Advances in molecular genetics of Marfan syndrome and related disorders

**DOI:** 10.3389/fgene.2026.1820063

**Published:** 2026-03-30

**Authors:** Georgia Damoraki

**Affiliations:** 4th Department of Internal Medicine, Department of Medicine, School of Health Sciences, National and Kapodistrian University of Athens, Athens, Greece

**Keywords:** case report, fibrillin-1 gene, Marfan syndrome, molecular genetics, novel FBN1 variants

Marfan syndrome (MFS, OMIM #154700) is a rare heritable multisystem connective tissue disorder (HCTD) with an estimated incidence of 1 in 3,000–5,000 individuals. The syndrome is characterized by a variable combination of cardiovascular (major blood vessels and heart valves), musculoskeletal, ocular (e.g., ectopia lentis and myopia), and pulmonary manifestations leading to variable phenotypic expression. It is inherited in an autosomal dominant pattern. The disease occurs worldwide, with no preference for race or gender. In 90% of cases, it is caused by mutations in the fibrillin-1 (*FBN1*, OMIM #134797) gene on chromosome 15q21.1, a major component of extracellular matrix (ECM) microfibrils. This leads to structural weakness of connective tissue and abnormal regulation of transforming growth factor beta (TGF-β) signaling. Revised Ghent Nosology (Loeys et al., 2010), in conjunction with genetic analysis of *FBN1* using omics technologies, contributes significantly to the diagnostic process. Despite progress, the complexity of MFS’s phenotypic variability and its molecular pathogenesis remains partially understood.

The Frontiers Research Topic entitled “*Advances in molecular genetics of Marfan syndrome and related disorders*,” comprising one original and three case report articles, underscores the evolving landscape of MFS and *FBN1*-related disorders via novel research findings. In addition, these studies highlight advances in genetic discovery and diverse clinical presentations, paving the way for improved risk stratification, diagnosis, consultation, and personalized management for patients worldwide.

The first article is a case study, in which Chen et al. summarize the difficulties regarding the diagnostic and therapeutic experience of geleophysic dysplasia **(**GD) complicated by pulmonary hypertension (PH) of a rare, complex case of a 2-year-3-month-old Chinese male patient with *FBN1* mutation-associated GD, alongside severe PH and progressive mitral stenosis with poor outcome. Genetic testing confirmed a heterozygous *de novo* pathogenic *FBN1* mutation c.5096A>G (p.Tyr1699Cys) in exon 42. *FBN1* mutation-related GD can produce complex PH driven by both pre- and postcapillary mechanisms, as well as progressive lung disease. Only a few cases of GD complicated by PH have been reported. Current therapeutic methods, like a prostacyclin analog (treprostinil) and mitral valvuloplasty, could provide short-term improvement but do not prevent progression if valve pathology is severe. In such rare and challenging cases, multidisciplinary management, molecular testing, and early surgical intervention, before advanced pulmonary or respiratory disease develops, may improve prognosis.

The second case study article is the first study in South Africa to identify MFS patients by using molecular techniques. Mhlongo et al. screened for *FBN1* gene mutations in a cohort of 14 South African patients with suspected or confirmed MFS using targeted next-generation sequencing (tNGS). Three individuals with suggestive clinical features lacked detectable *FBN1* mutations, emphasizing the diagnostic challenges in resource-limited settings. Seven likely pathogenic or pathogenic *FBN1* variants (five previously reported and two novel variants) were identified in 11 patients. Some variants were located in exons associated with severe, early-onset forms of MFS. The study advocates for integrating *FBN1* molecular testing into the standard of care for MFS in Africa to enable early diagnosis, improved management, and family counseling.

In another case report article, Marsan et al. describe a novel heterozygous missense variant (c.2348A>G, p.Ser783Arg) of the *FBN1* gene, in exon 20, which had not yet been reported in genetic databases, observed in a Sardinian family. The variant, which was first identified in a pediatric proband with characteristic clinical features (Revised Ghent Nosology) and subsequently validated by high-throughput sequencing (HTS), was confirmed in other family members with symptoms of MFS across four generations but not in those without symptoms. The finding supports an autosomal dominant inheritance pattern and demonstrates complete penetrance. The affected individuals displayed varying phenotypes. Based on the ACMG/AMP variant scoring system, the variant was classified as “likely pathogenic.” This discovery of the novel *FBN1* variant could help health professionals better understand how different gene mutations cause the disease and underscores the importance of genetic testing and family screening in the MFS diagnosis and management.

Finally, another important contribution expanding the known mutation spectrum of *FBN1* and underscoring the importance of functional validation in genetically unresolved MFS cases is the original research study by Wang et al. The analysis demonstrated the presence of a novel, *de novo* deep intronic variant, which was predicted to disrupt RNA splicing, in intron 40 of the *FBN1* gene (c.4943-8_4943-7insTATGTGATATTCATTCAC) in a 2-month-old Chinese female infant clinically and molecularly diagnosed with neonatal MFS (nMFS) and the absence of the variant in her parents. Τhe mutation caused exon 41 skipping, resulting in the deletion of 41 amino acids from the fibrillin-1 protein. That was verified by a minigene splicing assay. The study emphasizes that deep intronic variants could be pathogenic and highlights the importance of functional validation of such variants in undiagnosed MFS cases.
